# A Hypothesis-Based Framework for Chicken Meat Palatability: Proposing Indirect Roles of Arachidonic Acid and Lipid Oxidation

**DOI:** 10.3390/ani16121844

**Published:** 2026-06-15

**Authors:** Hideaki Takahashi

**Affiliations:** National Agriculture and Food Research Organization (NARO), Tsukuba 305-8517, Ibaraki, Japan; takahashi8675@gmail.com; Tel.: +81-80-5497-3127

**Keywords:** chicken meat palatability, Jidori, arachidonic acid, kokumi, calcium-sensing receptor, lipid oxidation, taste modulation, poultry science

## Abstract

Chicken meat palatability is shaped by what the meat contains (e.g., intramuscular fat and fatty-acid composition), what happens to those components during storage and cooking (including oxidation and transfer into soups or meat juices), and how tastebud signaling integrates the resulting stimuli. Chicken sold in Japan as “Jidori” (premium native-line products) is often described as having a richer flavor that lingers longer than that of standard broiler chicken, and published poultry work, including reports by the author and their colleagues, has linked this phenotype to higher arachidonic acid (AA) levels in the meat; however, the mechanistic basis remains under debate and has not been overturned. In addition, intact AA is a highly hydrophobic long-chain fatty acid that partitions poorly into aqueous phases, making a direct “AA-as-tastant” mechanism unlikely. This review develops a hierarchical interpretation that separates food-level associations from tastebud mechanisms and reframes AA as a primarily downstream lipid substrate. Two complementary routes are proposed: (i) a food-chemistry route in which cooking and storage oxidation generate low-molecular-weight, water-accessible lipid-oxidation products that partition into soups, meat juices, and cooking loss, and (ii) a receptor-centered route in which kokumi-related signaling pathways, particularly those involving the calcium-sensing receptor (CaSR), amplify taste intensity, continuity, and aftertaste within tastebuds. This framework emphasizes how these routes can be linked experimentally by combining matrix/phase manipulations with targeted carbonyl profiling, fractionation–reconstitution, and pathway-perturbation assays in tastebud readouts. Overall, the model is intended to support mechanism-focused study designs beyond single-compound explanations.

## Simple Summary

In Japan, native chicken breeds are used to produce premium products sold under the “Jidori” certification scheme. Consumers often describe Jidori chicken as tasting richer and having a more pleasant aftertaste compared with standard broiler chicken. Several studies have linked this sensory difference to the fat in the meat, especially a fatty acid called arachidonic acid (AA). However, AA itself is a large, oily molecule and unlikely to be detected directly by taste receptors. The more plausible explanation is an indirect one. During storage and cooking, fat components can be transformed into smaller compounds that dissolve into soups and meat juices and can reach the tastebuds. This review summarizes the evidence supporting this idea and proposes a simple two-step way to think about chicken meat palatability. First, the composition of the meat and the way it is handled and cooked determine which small, water-accessible compounds are formed and released. Second, some of these compounds may strengthen and prolong taste signals, particularly in umami-rich foods, making the taste seem more continuous and satisfying. From a practical viewpoint, this means that cooking methods and whether fat is retained or removed can change what reaches the mouth and, ultimately, what people perceive. The goal of this review is to provide a clear starting point for experiments that link measurable chemical changes in cooked chicken to sensory perception.

## 1. Introduction

Chicken from slow-growing native breeds is frequently described as having a deeper and more persistent flavor than standard broiler meat—even when levels of classic umami contributors such as free amino acids and inosine 5′-monophosphate (IMP) are comparable [1]. In Japan, premium native chicken is marketed under the JAS-certified Jidori scheme, which was established to promote the use of indigenous Japanese breeds. Under the Japanese Agricultural Standard (JAS), Jidori refers to chicken meat from birds with at least 50% native-breed ancestry, reared for a minimum of 75 days and kept on floor systems from day 28 onward under specified stocking-density criteria [2]. Such products are consistently characterized by greater richness and continuity and a superior aftertaste compared with commercial broilers [2,3]. Across poultry studies, one compositional feature appears repeatedly: Jidori and other native-breed meats tend to contain higher levels of arachidonic acid ((AA) 20:4n-6), and elevated AA content is associated with a stronger sensory preference and kokumi-like descriptors [1,4]. The challenge is to move from this robust association to a mechanistic explanation that can be tested experimentally.

Dietary intervention studies reinforce this link: using AA-enriched oil in feed increases tissue AA levels and improves sensory scores for both broilers and native chickens [5,6]. These findings are sometimes summarized as “more AA means better taste,” yet the underlying mechanism remains unclear because intact AA is a long-chain, strongly hydrophobic fatty acid, unlike the small, polar molecules that typically act as tastants in the oral aqueous phase, and direct orthosteric binding of AA to kokumi- or umami-related receptors has not been demonstrated in taste systems [7,8]. Given these constraints, AA is better conceptualized as a downstream lipid precursor whose sensory impact emerges only after receptor-linked mobilization and conversion into signaling-active lipid mediators. More broadly, lipid oxidation in meat is a multistage process that converts unsaturated fatty acids into hydroperoxides, aldehydes, ketones, alcohols, and other secondary products, with consequences for both desirable flavor generation and quality deterioration [9]. To avoid conflating food-level observations with receptor-level plausibility, this review separates two questions: (1) which cooking, storage, and matrix conditions generate lipid-derived species capable of entering the oral aqueous phase, and (2) how does tastebud signaling shape intensity and persistence once these species reach the mouth? From a food-chemistry perspective, AA is treated primarily as a substrate yielding low-molecular-weight oxidation products during storage and heating, a subset of which can partition into soups, meat juices, and cooking loss and plausibly come into contact with tastebuds. From a physiological perspective, kokumi is discussed in relation to the calcium-sensing receptor (CaSR), a class C GPCR reported in tastebud cells, together with within-bud amplification mechanisms influencing thickness, mouthfulness, continuity, and aftertaste [7,8].

This review is presented as a hypothesis-driven narrative synthesis that is designed to build a mechanistic framework rather than provide exhaustive, systematic coverage. To enhance transparency in how the literature was identified, relevant studies were gathered through targeted searches of major scientific databases (e.g., PubMed and Web of Science) using combinations of terms such as “chicken,” “palatability,” “arachidonic acid,” “lipid oxidation,” “kokumi,” and “CaSR.” These searches were supplemented by citation tracking of key primary papers and reviews. Articles were selected for inclusion based on their conceptual and mechanistic relevance to the proposed framework, with particular emphasis on studies offering biochemical, sensory, or receptor-level evidence.

## 2. Arachidonic Acid as a Compositional Marker Associated with Chicken Meat Palatability

### 2.1. Arachidonic Acid Variation in JAS-Certified Jidori Chickens and Its Sensory Implications

Comparative analyses show that JAS-certified Jidori chickens contain higher levels of AA than commercial broilers, reflecting consistent differences in fatty-acid composition across multiple Japanese native lines used for Jidori production [2,4]. Controlled feeding studies further demonstrate that increasing AA content enhances sensory preference even when other flavor contributors such as free amino acids and IMP remain unchanged [5,6]. In both Hinai-jidori and broilers, supplementation with AA-enriched oil improves umami, kokumi, and aftertaste in a dose-dependent manner under within-line paired designs [5,6]. Complementary analyses of Korean native chicken have identified AA as a major fatty-acid component and suggest that, together with classic taste-active compounds such as IMP, it contributes to perceived quality differences, with bird age and cooking conditions influencing measured levels [10]. Collectively, these findings indicate that AA is an independent compositional factor contributing to chicken meat palatability beyond what traditional umami-related components alone can explain [5,6,10].

### 2.2. Genetic Regulation of Arachidonic Acid Content

AA content in chicken meat is shaped not only by diet and rearing conditions but also by genetic factors [2,4,11,12]. Polymorphisms in fatty-acid-metabolism genes—particularly within the fatty acid desaturase (FADS) cluster—are associated with variation in AA content, reflecting their role in converting linoleic acid (LA) into longer-chain PUFAs, including AA [11,12]. This genetic control links poultry-genetics research to meat palatability, and AA has therefore been regarded as a measurable trait and potential marker for explaining meat-quality differences among genotypes [2]. This framework helps clarify why Japanese native chicken lines used for Jidori production—lines not intensively selected for rapid growth—retain flavor characteristics distinct from those of commercial broilers [4]. These AA-related differences are therefore treated as a compositional trait relevant to palatability within JAS-defined production contexts, and this trait is revisited in later sections where AA is mechanistically reinterpreted as a downstream lipid precursor rather than a direct taste contributor.

### 2.3. Unresolved Mechanistic Questions

In this section, the reported AA–palatability relationship is interpreted as being conditional upon cooking and handling conditions and the evaluated matrix, unless broader generality is explicitly demonstrated [2]. Although several studies report a positive association between AA content and chicken meat palatability, the strength and transferability of this relationship across products and protocols remain uncertain, and even intervention designs (e.g., AA-enriched oil feeding) have not identified the molecular link between AA and taste perception [2,5,6]. The key unresolved issue is whether AA directly engages kokumi- or umami-related receptor pathways or instead contributes indirectly through downstream chemical or physiological mechanisms—an interpretation difficult to reconcile with established taste-receptor biology (Section 3) [7,8]. Some AA-linked sensory differences may be phase-sensitive—appearing in fat-containing soups or broths and reduced by defatting—because the lipid phase can retain hydrophobic precursors, promote carbonyl formation during heating, and mediate transfer into the aqueous fraction. Accordingly, both fat-containing and defatted matrices should be evaluated, noting that fat can also increase perceived richness via oral-fat perception and mouthfeel (Section 8.1).

## 3. Limitations of a “Direct Taste Ligand” Interpretation of Arachidonic Acid

Although AA content consistently correlates with chicken meat palatability, a “direct taste-ligand” interpretation is difficult to reconcile with established taste-receptor biology. Intact AA is a long-chain, highly hydrophobic PUFA that partitions poorly into the oral aqueous phase, lacks structural compatibility with the polar-ligand binding mode of class C GPCRs such as CaSR and T1Rs, and has no reported orthosteric binding activity in taste systems. Consistent with this partitioning logic, aqueous fractions from oxidized AA preparations contain low-molecular-weight, water-accessible oxidation products rather than the parent fatty acid. Within cells, AA functions mainly as a downstream substrate released by PLA_2_ and converted into lipid mediators following Ca^2+^ mobilization, a biology incompatible with intact AA acting as a primary tastant. Therefore, AA is treated here as a food-level compositional correlate and a precursor to water-phase-accessible oxidation products, while tastebud mechanisms are attributed to downstream modulators such as oxidized-AA-derived carbonyls and CaSR-centered kokumi pathways.

### 3.1. Oxidized Arachidonic Acid as a Gustatory Modulator of Umami

Oxidized AA must be interpreted within workflow-specific contexts because the composition of oxidation products depends on heating, the matrix microenvironment, and extraction/handling conditions. If AA contributes to kokumi or umami, the most plausible mediators are not intact AA but water-accessible oxidation products generated during mild oxidation or thermal processing [13,14]. This yields a direct prediction: under matched processing conditions, AA-rich, fat-containing matrices should produce aqueous fractions enriched in low-molecular-weight carbonyl mixtures that enhance umami-related readouts (and CaSR-centered modulation) more strongly than LA-rich matrices, whereas defatting should attenuate the effect [13,14].

#### 3.1.1. AA Autoxidation Generates Diverse Secondary Product Classes

AA is highly susceptible to autoxidation and thermal degradation because of its multiple double bonds. Under mild oxidation, AA yields a mixture of hydroxy-alkenals (e.g., 4-hydroxy-2-nonenal), dialdehydes, and short-chain carbonyls that can influence flavor [15,16,17,18,19].

#### 3.1.2. Partitioning into the Aqueous Phase: Why the “Active Fraction” Is Not Intact AA

Because intact long-chain PUFAs are effectively water-insoluble, oxidation in lipid-rich tissue produces mixtures in which only small, polar, water-accessible products can migrate into meat juices, drip/cooking loss, soups, or broths [20]. Kiyohara-type aqueous extracts therefore enrich low-molecular-weight oxidation products rather than parent AA or LA, and the precise composition of this aqueous fraction depends on oxidation conditions, matrix environment, and handling workflows. This partitioning logic underlies why the “active fraction” in oxidized-AA taste-modulation studies reflects water-accessible carbonyls rather than intact PUFAs.

#### 3.1.3. Analytical Workflows: Volatile vs. Non-Volatile Products

Volatile carbonyls are typically profiled using HS-GC or HS-SPME-GC, whereas more polar or less volatile products in aqueous extracts are analyzed via LC–MS [21,22,23]. Volatile outputs track aroma-side chemistry, while gustatory modulation is restricted to compounds present in—or transferable to—aqueous fractions. Thus, aroma markers such as 2,4-DD are not primary candidates for taste-active aqueous mechanisms. AA–LA differences are interpretable only under matched cooking and capture workflows [22,23].

#### 3.1.4. Working Map for Candidate Selection: Solubility-Based Classification

A solubility-based classification (Table 1) provides a qualitative framework for selecting candidate compounds in Kiyohara-type aqueous-extract assays [13,14]. Table 1 summarizes which oxidation-product classes are expected to appear in aqueous extracts and highlights that long-chain aldehydes (e.g., 2,4-DD) are unlikely to be enriched except via emulsion/protein-bound carryover.

##### Purpose and Scope of Table 1

Table 1 is a solubility- and partitioning-based working map for interpreting Kiyohara-type aqueous-extract assays [13,14]. It is not claimed to be capable of determining which compounds dominate in every meat matrix. The cited references support the underlying partitioning logic, representative compound classes, and interpretation limits rather than asserting universal quantitative dominance.

##### Assumptions and Caveats to Consider When Interpreting “Water Extracts”

Interpreting “water extracts” requires three practical cautions. First, “aqueous” does not mean lipid-free: trace emulsified lipids can carry hydrophobic aldehydes or oxylipins into the fraction [9], and the observed abundance reflects both formation and partitioning. Therefore, compounds should be measured directly in the aqueous fraction rather than inferred from polarity alone [9]. Second, “detected” does not necessarily mean “free”: carbonyls can reversibly bind to proteins, amino acids, or thiols, reducing the bioavailable concentration relative to the analytical total [9]. Third, “measured” does not necessarily mean “retained”: the most volatile carbonyls can be lost during incubation or handling [9,24], creating a handling-loss channel. Even with these caveats, the key conceptual point remains that the Kiyohara-type aqueous-extract assay [13,14] enriches water-accessible, low-molecular-weight oxidation products rather than intact PUFAs, strengthening the interpretation that the observed enhancement is mediated by oxidation products.

##### Evidence Base Supporting Candidate Classes (Including AA-Specific Oxidation Products)

The candidate classes in Table 1 align with established lipid-oxidation frameworks [9,16,24] and the Kiyohara-type aqueous-extract context [18]. Low-molecular-weight carbonyls are the most evidence-supported gustatory modulators because their effects persist under olfactory deprivation and can be mediated via orally expressed CaSR (Section 3.1.5) [7,13,14,25]. One AA-derived product is (E,Z,Z)-2,4,7-tridecatrienal (TAG), which is an AA-specific oxidation marker because its formation requires the C20 backbone and bis-allylic arrangement unique to AA; LA does not generate this triene aldehyde under comparable conditions [18,19]. TAG is useful for tracking AA-linked oxidation pathways, but it is not considered a leading taste-active species.

AA autoxidation datasets (from AEDA and isotope-dilution analyses) provide analytical targets, including hexanal; 1-octen-3-one; (E,Z)/(E,E)-2,4-DD; trans-4,5-epoxy-(E)-2-decenal; and TAG [9,18,19]. As a working hypothesis, LA oxidation tends to yield a more C6-biased profile (e.g., hexanal), whereas AA oxidation yields a more distributed C3–C5 + C6 mixture [17,18,19]. Because CaSR PAM efficacy varies across aldehydes, a more diverse short-chain aldehyde mixture could provide broader subthreshold stabilization of CaSR activation, potentially contributing to AA>LA taste enhancement under controlled conditions [26]. This can be tested by profiling individual C3–C6 aldehydes in matched AA vs. LA aqueous fractions and reconstituting mixtures in measured ratios in CaSR assays and tastebud readouts [14,26].

##### Practical Roadmap for Chemistry–Function Linking

The identity and causal contribution of the water-extractable “active principle(s)” remain unresolved [13,21,22]. The next steps include targeted profiling within the aqueous fraction using complementary workflows—HS-GC/GC–MS with stable-isotope dilution for prioritized carbonyls and LC–MS/MS for polar/non-volatile products—paired with fractionation–reconstitution tests for necessity and sufficiency. For meat-science relevance, candidate modulators should be quantified directly in consumer-relevant aqueous matrices (soups, broths, meat juices, and cooking loss) and reported in aqueous-phase units (e.g., μM or μg/L) alongside fat content and processing conditions [27,28]. “Water-extractable” does not imply “fat-independent,” because phase/matrix sensitivity can reflect upstream lipid precursors and oral-fat cues (Section 8.1).

#### 3.1.5. Evidence Consistent with Gustatory (Not Merely Olfactory) Modulation

Although lipid-oxidation products are often treated as aroma contributors [29,30,31], multiple lines of evidence indicate that oxidized AA preparations can modulate taste independently of olfaction. In anosmic mice, aqueous extracts from autoxidized AA ethyl ester altered licking responses to tastants and added hexanal modified responses to quinine and MSG within the same workflow [21]. Gustatory electrophysiology showed that oxidized AA and hexanal enhanced responses to MSG, supporting a peripheral gustatory contribution [19]. These findings support viewing short-chain aldehydes and related carbonyls as practical starting points for chemistry–function linking in meat-relevant aqueous fractions.

#### 3.1.6. Intracellular Signal-Amplification Hypothesis (Ca^2+^–TRPM5)

##### Positioning This Route Within the CaSR-Centered Route

This intracellular-amplification view aligns with CaSR-centered kokumi signaling, in which receptor-dependent Ca^2+^ mobilization and downstream free-AA release via PLA_2_ can prolong within-bud modulation. CaSR activation produces sustained Ca^2+^ dynamics that feed into the same Ca^2+^–TRPM5 node described above, providing a mechanistic link between kokumi-related descriptors (thickness, mouthfulness, and continuity) and oxidized-AA-derived modulators. In this integrated framework, CaSR-mediated Ca^2+^ mobilization and carbonyl-driven Ca^2+^–TRPM5 gain act as convergent routes that enhance the temporal and intensity dimensions of umami perception without altering taste quality.

##### Food-Focused, Testable Predictions

This hierarchical model yields three food-focused, testable predictions:

1. Matrix/phase dependence

The AA-linked “taste intensity” advantage is expected to be strongest in fat-containing soups or broths, attenuated by defatting and partially restored by reconstitution with the corresponding lipid fraction (or its controlled cooking-derived products) [4,5,10].

2. Aqueous-phase carbonyl covariation

In the aqueous fraction, candidate low-molecular-weight carbonyls (e.g., C5–C6 aldehydes) are expected to covary with sensory enhancement and/or CaSR-bioassay outputs. Fractionation followed by reconstitution at measured concentrations provides a direct way to reproduce—or falsify—the phenotype [19,21].

3. Pathway sensitivity

Enhancement is expected to be Ca^2+^-dependent and to decrease with CaSR inhibition and/or within-bud cholinergic blockade, consistent with amplification of Ca^2+^–TRPM5-linked dynamics rather than a change in taste quality [7,8,19,20,22].

These food-focused predictions reappear in later sections, where they are integrated with tastebud signaling and sensory persistence to form a broader mechanistic framework.

## 4. Roast-Aroma Chemistry in Cooked Chicken: 2,4-DD Formation from LA and AA

Section 4 summarizes roast-aroma chemistry in cooked chicken, using 2,4-decadienal (2,4-DD) as a practical marker compound. The aim is to explain how n-6 PUFA composition—particularly the balance between linoleic acid (LA; 18:2n-6) and arachidonic acid (AA; 20:4n-6)—can shift volatile outputs under defined cooking and capture conditions, without implying that 2,4-DD alone determines palatability [17,30,32]. Interpretation is strongly protocol-bounded: workflow-specific effects arise from heating and volatile-capture settings, whereas context-specific effects reflect matrix factors such as lipid class, water activity, catalysts/antioxidants, oxygen exposure, and sinks. Because oxidation reflects competing pro-oxidant and antioxidant processes, reported LA/AA → 2,4-DD relationships should be treated as hypotheses unless cooking and capture workflows are matched [33].

2,4-DD has repeatedly been identified as a key odorant contributing roasted, fried, and chicken-like notes in heated poultry lipids [24,30]. It arises from n-6 PUFA oxidation (LA and AA) via hydroperoxide formation followed by β-scission and is treated here as an aroma-side marker distinct from the taste-active aqueous fraction emphasized in Section 3.1 [16,32,33].

### 4.1. Overview of Lipid Oxidation Pathways in n-6 Fatty Acids

The specific hydroperoxide species formed from LA and AA, their positional selectivity, and the competing fragmentation channels differ between the two fatty acids and are strongly shaped by heating conditions and matrix factors. These differences influence the relative formation of C6 aldehydes versus C10 dienals under defined cooking workflows. This section provides only the mechanistic context needed to interpret genotype- or diet-linked differences under the workflow-specific conditions described in Section 4.

#### 4.1.1. Oxidation of LA

LA oxidation is commonly summarized as LA → 9-/13-HpODE → β-scission products [16,24]. Under many heating regimes, the 13-HpODE route can yield both C6 aldehydes (e.g., hexanal) and a C10 dienal (2,4-DD) [16,24,32], and the apparent balance between these channels depends on cooking conditions and the stability of the specific hydroperoxides formed. In practical poultry systems, LA-rich matrices often show a stronger C6 bias, with 2,4-DD formation increasing only under heating conditions that favor 13-HpODE fragmentation [16,24,32].

#### 4.1.2. Oxidation of AA

AA contains more double bonds and bis-allylic positions than LA, producing a broader set of hydroperoxides and hydroxy-alkenal precursors that can fragment through multiple β-scission channels [16,24,27]. As a result, AA oxidation tends to generate a more distributed mixture of C3–C5 aldehydes in addition to C6 species [16,25,27], with 2,4-DD formation depending strongly on heating regime and matrix factors [18]. These AA-specific fragmentation patterns provide a mechanistic basis for differences in volatile profiles between AA-rich and LA-rich systems under matched workflows.

#### 4.1.3. Comparative Perspective: LA vs. AA

The comparative fatty-acid data are summarized in Table 2, which compares the levels of linoleic acid (LA) and arachidonic acid (AA) between Hinai-jidori chickens and broilers raised under identical feeding conditions and rearing periods. Under matched workflows, LA and AA can bias aldehyde profiles differently because they differ in terms of oxidation susceptibility, hydroperoxide stability, and fragmentation allocation [16,24,30,32]. LA-rich systems typically show a stronger C6 aldehyde contribution, whereas AA-rich systems yield a more distributed C3–C5 + C6 mixture [16,18,27]. AA-rich matrices can generate higher levels of 2,4-DD only under heating regimes that favor the relevant fragmentation channels, reflecting the strong protocol dependence of dienal formation [18,30,32]. These tendencies are heuristic rather than universal and must be validated empirically within each cooking and capture protocol.

### 4.2. β-Scission Mechanisms Leading to 2,4-DD

Hydroperoxide β-scission proceeds through O–O homolysis, forming an alkoxyl radical, followed by C–C cleavage that yields aldehyde fragments. The efficiency and product distribution of this pathway depend on hydroperoxide position, double-bond conjugation, and the stability of the resulting radical intermediates [17,18,24,30,31]. These structural factors determine whether a given hydroperoxide can generate a C10 dienal fragment consistent with 2,4-DD formation. The subsequent Section 4.2.1, Section 4.2.2 and Section 4.2.3 outline how these mechanistic principles apply to LA- and AA-derived hydroperoxides under defined heating conditions.

#### 4.2.1. LA-Derived β-Scission Pathways

In LA-derived systems, β-scission from 13-HpODE is the primary route capable of generating a C10 dienal fragment consistent with 2,4-DD formation [17,18,24,30]. The efficiency of this pathway depends on the stability of the 13-alkoxyl radical and the conjugation pattern formed after O–O cleavage. Under typical poultry-relevant heating regimes, 13-HpODE fragmentation more commonly yields C6 aldehydes such as hexanal, with 2,4-DD arising only when thermal conditions favor extended conjugation and C10-fragment retention [16,24,32].

#### 4.2.2. Alternative LA Fragmentation Channels

LA can also form 9-HpODE, whose β-scission products predominantly yield shorter aldehydes (e.g., pentanal) rather than C10 fragments [17,24,32]. These alternative channels illustrate why LA-rich matrices typically show a strong C5–C6 aldehyde signature and why 2,4-DD formation from LA is restricted to specific thermal conditions that stabilize extended conjugation in the 13-HpODE pathway [17,24,32].

#### 4.2.3. AA-Derived β-Scission Pathways

AA contains additional double bonds and bis-allylic positions, producing hydroperoxides (e.g., 12-HpETE) that can undergo β-scission and thus yield a broader distribution of aldehydes [16,27]. Fragmentation of 12-HpETE can generate a C10 dienal fragment under heating regimes that stabilize the corresponding alkoxyl radical and conjugated intermediates [16,18]. Because AA-derived hydroperoxides access multiple β-scission geometries, AA-rich matrices often show a more diverse C3–C5 + C6 aldehyde mixture, with 2,4-DD formation remaining strongly dependent on the specific thermal and matrix conditions applied [16,18,27].

### 4.3. Literature Survey: Fatty-Acid Composition vs. 2,4-DD in Poultry

Across poultry studies using comparable heating and volatile-capture workflows, differences in fatty-acid composition—particularly the relative abundance of LA and AA—are associated with systematic shifts in measured aldehyde profiles. Model-system work conducted by Elmore and colleagues revealed that AA-containing substrates can yield higher levels of 2,4-DD under specific thermal conditions, whereas LA-rich substrates more consistently generate C6 aldehydes such as hexanal [17,30,34]. Poultry-lipid studies show similar tendencies: samples with higher AA/LA ratios often exhibit greater 2,4-DD abundance, while LA-skewed samples display stronger C6 signatures [1,9,16,17]. Although the magnitude of these differences varies across studies, the directional pattern is consistent—AA-rich systems are more capable of producing detectable dienals under defined cooking conditions. Overall, the literature supports a conditional association between fatty-acid composition and 2,4-DD output, without implying that 2,4-DD alone determines poultry flavor.

### 4.4. Relevance to Poultry Flavor Chemistry

For poultry flavor chemistry, the practical value of 2,4-DD lies in its role as a reproducible marker of roast-aroma formation rather than as a determinant of palatability. Studies comparing poultry lipids with differing fatty-acid compositions show that measured dienal levels reflect the combined influence of substrate composition, heating regime, and analytical capture and therefore serve as indicators of how n-6-PUFA-derived pathways are expressed under specific cooking conditions [9,25,27,30,31]. When interpreted alongside broader aldehyde profiles, 2,4-DD helps contextualize how LA- and AA-linked oxidation routes contribute to the volatile pathway of cooked-chicken flavor. This marker-based perspective supports the integration of compositional data with controlled cooking workflows, clarifying how lipid-derived volatiles participate in poultry flavor development.

### 4.5. Applications in Poultry Studies and Flavor Design

In poultry studies, the AA/LA balance can be used as a practical stratification variable for designing experiments that link compositional differences to measurable chemical outputs. When combined with standardized cooking and volatile-capture workflows, this approach enables the performance of reproducible comparisons across genotypes, diets, or processing conditions. AA-rich and LA-rich matrices can therefore be positioned as mechanistic contrasts that help clarify how lipid-derived pathways contribute to cooked-chicken flavor. This stratified design supports coordinated evaluation of volatile profiles, aqueous-phase chemistry, and sensory outcomes without re-specifying the underlying tastebud mechanisms addressed in Section 3.

### 4.6. Proposed Heuristic Model for 2,4-DD Formation from n-6 PUFA Composition

#### 4.6.1. Conceptual Framework

The proposed framework integrates food-chemistry pathways with tastebud signaling to explain how lipid composition and processing conditions shape chicken meat palatability. At the food level, cooking and storage determine which low-molecular-weight lipid-derived species become available in volatile and aqueous phases. At the sensory-physiology level, these species interact with established within-bud amplification pathways, including kokumi-related signaling, to influence intensity, continuity, and aftertaste. The framework is designed to separate compositional inputs, processing-dependent chemical outputs, and tastebud responses into analytically tractable components, providing a structured basis for experimental validation in poultry systems.

#### 4.6.2. Mechanistic Basis for the Directional Assumption

The directional assumption is grounded in the structural factors summarized in Table 3, which outline how differences in initiation susceptibility, hydroperoxide stability, and fragmentation allocation can shift the relative efficiency of LA- and AA-derived pathways under specific heating and capture conditions. These factors do not imply universal dominance of one precursor over the other; rather, they provide a mechanistic rationale for expecting systematic but workflow-dependent differences in dienal-related outputs across poultry matrices [15,25,29,31].

### 4.7. Future Directions

Future studies should test the AA/LA-weighted heuristic under standardized cooking and volatile-capture workflows by pairing composition measurements with targeted 2,4-DD quantification and sensory evaluation. Broader profiling of co-varying aldehydes and oxidation intermediates will help clarify how lipid-derived pathways are expressed across poultry matrices. Comprehensive reporting of processing and analytical parameters will be essential for reproducibility and cross-study comparison [1,29,30,32,35].

## 5. Calcium-Sensing Receptor and Kokumi: A Mechanistically Developed Candidate Pathway for Taste Modulation

### 5.1. Concept of Kokumi and Its Sensory Characteristics

Kokumi denotes sensory qualities that enhance the perceived depth and continuity of existing tastes rather than forming a distinct taste category. It is typically described using terms such as continuity, mouthfulness, and thickness, and it has been reported to be triggered by a diverse array of foods, including fermented products, soups, and meat-based dishes [6]. Kokumi sensations become especially apparent when kokumi-active compounds occur alongside umami contributors such as glutamic acid or IMP; under these conditions, they amplify and prolong the underlying taste profile [6,7]. In practical sensory terms, kokumi is best understood as an amplification layer that increases the perceived richness and persistence of an existing flavor.

### 5.2. Expression and Functional Role of CaSR in Tastebuds

Among proposed kokumi-related receptors, the calcium-sensing receptor (CaSR) has the strongest mechanistic support [6,7,8,36]. CaSR is a class C GPCR first characterized for systemic calcium homeostasis [35], and later work showed that it is expressed in subsets of tastebud cells that are distinct from cells expressing canonical sweet or umami receptors [7,8]. This cellular segregation is consistent with a modulatory role in tastebuds rather than primary taste coding. Importantly for poultry research, oral expression and functional analyses of CaSR have also been reported in relation to chickens, supporting the plausibility of CaSR-centered kokumi mechanisms in avian taste biology [37]. In addition to early human work, functional studies on mouse taste cells have demonstrated CaSR activity in response to kokumi substances [7,8], and activation of CaSR in tastebud cells induces intracellular calcium signaling that modulates neural responses to basic taste stimuli [7,8,36].

### 5.3. γ-Glutamyl Peptides as Kokumi Substances Acting on CaSR

CaSR is the most consistently supported molecular candidate for kokumi-related modulation in tastebuds [6,7,8,36]. It is expressed in subsets of taste cells that are distinct from those expressing canonical sweet or umami receptors, consistent with a modulatory rather than primary coding role [7,8]. Functional responses to kokumi-active compounds have been demonstrated in mammalian taste cells [7,8], and CaSR activation influences intracellular calcium dynamics relevant to taste-signal amplification [7,8,36]. For poultry applications, reports of CaSR expression and functional activity in the oral tissues of chickens provide a basis for considering CaSR-centered kokumi mechanisms within avian taste biology [37].

### 5.4. Structural Basis of CaSR Activation Revealed by Cryo-EM Analysis

Recent cryo-EM work has provided direct structural evidence of kokumi-peptide recognition by CaSR. A 2025 structural study captured CaSR bound to γ-Glu-Val-Gly, revealing a well-defined orthosteric pose within the VFT domain and an associated closed-VFT conformation linked to receptor activation [38]. The peptide is stabilized by multiple polar and electrostatic contacts, and the observed VFT closure offers a structural route for propagating ligand binding toward the transmembrane domain. These findings complement earlier functional studies by demonstrating that γ-glutamyl peptides can directly engage the CaSR orthosteric site. At the same time, structural data alone do not establish kokumi perception; sensory and pharmacological evidence remains essential for linking CaSR activation to kokumi-related modulation [6,7,8,39]. CaSR’s ability to integrate co-agonists such as extracellular Ca^2+^ and exhibit context-dependent signaling further helps explain the variability in kokumi enhancement across foods and experimental designs [14,36,38,39].

### 5.5. Cooperative and Amplification Mechanisms Within Tastebuds

Within tastebuds, kokumi-related modulation is understood as an amplification process in which CaSR activation enhances the persistence and perceived depth of ongoing taste-evoked signals rather than generating a distinct taste quality. Cooperative interactions with extracellular Ca^2+^ contribute to this amplification, providing a physiological basis for the characteristic continuity, mouthfulness, and thickness associated with kokumi [6,7,8,36].

#### 5.5.1. Ex Vivo Evidence for Within-Bud Amplification

Ex vivo tastebud preparations provide operational evidence for within-bud amplification: CaSR activation during tastant stimulation enhances transmitter output in a muscarinic-dependent manner, and this enhancement is abolished by atropine in the reported systems [7,14]. These findings support the existence of a CaSR-linked modulatory module, although the underlying CaSR → ACh → muscarinic wiring remains unresolved at cell-type resolution. Outstanding lines of inquiry include which taste-cell subtypes release and receive ACh, how broadly this module generalizes across papillae, and how its engagement depends on stimulus context—issues that currently limit definitive circuit-level interpretation [7,14].

#### 5.5.2. Intracellular Free-AA Mobilization as a Secondary Tuning Layer

(1) Canonical CaSR signaling (AA-independent).
  Upon activation, CaSR couples to G proteins (Gq/Gi), activates phospholipase C (PLC), and drives inositol 1,4,5-trisphosphate (IP_3_)-mediated intracellular Ca^2+^ mobilization. These upstream steps represent the primary CaSR response and proceed without requiring AA as an initiating ligand.
(2) Why AA mobilization could matter for kokumi-like persistence and amplification.

Within the hierarchical model, free-AA mobilization is positioned as a secondary tuning layer that reshapes the time course and gain of tastebud output after CaSR-driven Ca^2+^ mobilization. This yields explicit expectations: (i) interventions that reduce free-AA production should compress prolonged or oscillatory Ca^2+^ dynamics and narrow the persistence window of transmitter output; (ii) COX-dependent conversion to prostaglandins (e.g., PGE_2_) should contribute to autocrine/paracrine reinforcement via EP receptors and cAMP/PKA signaling; and (iii) for a fixed upstream stimulus, downstream lipid signaling should increase the efficiency of transmitter output (ATP release and potentially other transmitters), thereby strengthening “continuity” and aftertaste-related readouts. These expectations motivated the development of the oxylipin-modulation concept and the pathway-perturbation tests outlined in Section 8.5.4 [28,40].

#### 5.5.3. Non-Synaptic Cholinergic Signaling Within Tastebuds (ACh → M3R) and Words of Caution

##### Proposed Within-Bud Cholinergic Amplification Circuit: Hypothesis

The working hypothesis is that kokumi-related CaSR activation engages a local, non-synaptic cholinergic module in which ACh released from a presynaptic-like subset of tastebud cells acts on neighboring muscarinic receptors to amplify tastant-evoked output [14]. Operationally, this framework predicts atropine-sensitive enhancement and concurrent evidence of ACh-related activity within the same preparation. Because the specific CaSR → ACh → muscarinic wiring remains unmapped at cell-type resolution, the circuit is best treated as a testable module rather than established circuitry. This arrangement provides a mechanistic interpretation for kokumi’s ability to strengthen continuity and aftertaste without generating a distinct basic taste [6,14].

##### Evidence Limits and Words of Caution

Current evidence supports the notion that there is a muscarinic-dependent amplification step downstream of CaSR activation in isolated tastebud preparations, but the underlying CaSR → ACh → muscarinic wiring remains unmapped at cell-type resolution [14]. Reports of atropine-sensitive enhancement and ACh-related activity provide operational support for a local cholinergic module, yet they do not identify the specific ACh-releasing cells, the dominant muscarinic subtype(s), or the generality of this arrangement across papillae [7,14]. For this reason, the main text treats the pathway as a local, non-synaptic, Ca^2+^-dependent amplification module and keeps all cell-type-resolved claims explicitly hypothesis-bounded until direct mapping becomes available [14].

#### 5.5.4. A Working Type III “Hub” Model for Gain and Persistence: Hypothesis

A hypothesis-level integration of the preceding evidence is as follows: CaSR-linked cholinergic modulation is implemented by a small Type III-like “hub” population that elevates local intra-bud Ca^2+^ tone and thereby increases the gain and persistence of Type II-cell ATP output without altering primary taste quality [14]. Testable predictions follow from this framework: (i) kokumi-evoked Ca^2+^ responses and/or ACh-related markers should localize to a small subset of cells consistent with a Type III phenotype and precede enhanced Type II-cell ATP release; (ii) atropine should reduce enhancement even when CaSR activation is preserved, consistent with a required muscarinic step; and (iii) manipulations that increase intra-bud Ca^2+^ tone should prolong ATP-output dynamics, whereas Ca^2+^ buffering should compress the persistence window [14].

## 6. Repositioning Arachidonic Acid as a Downstream Lipid Precursor

### 6.1. Scope: From Food-Level Chemistry to Intracellular Signaling

Section 3 addressed molecules that can act directly in the oral aqueous phase, including water-extractable oxidation products formed during cooking. One complementary perspective is intracellular: once kokumi-related receptors are activated, downstream signaling within tastebud cells can mobilize free AA as a lipid substrate. Section 6 therefore shifts from food-level chemistry to intracellular events that shape the time course and gain of tastebud output, with the core cascade outlined in Section 6.2 [6,7,28,36,40].

### 6.2. Core Cascade: CaSR → Ca^2+^ → PLA_2_ → Free-AA Mobilization

The intracellular cascade considered in Section 6 begins with CaSR-evoked Ca^2+^ signaling, which activates Ca^2+^-dependent PLA_2_ and mobilizes free AA from membrane phospholipids. The liberated AA then serves as a downstream lipid substrate that can be converted into bioactive mediators, including COX-derived products relevant to signal persistence [28,40]. This cascade provides the mechanistic basis for the secondary tuning processes discussed in the subsequent sections.

### 6.3. Free-AA Mobilization as a Secondary Tuning Layer

Increases in free AA quantities in CaSR-positive taste cells are an acute, downstream consequence of receptor-evoked Ca^2+^ signaling rather than a longer-term expansion of the esterified membrane AA pool, as addressed in Section 6.4. Within this framework, stimulus-evoked free-AA mobilization functions as a secondary tuning layer: the liberated AA serves as a substrate for downstream lipid-mediator pathways that can modify the duration and gain of tastebud output. Such mediators, including COX-derived products, provide plausible routes for sustaining or amplifying CaSR-initiated responses via autocrine or paracrine feedback [40]. When applied to taste, this supports the view that AA-derived metabolites act as signal shapers rather than discrete taste inputs.

### 6.4. AA Pool Expansion and Signal Gain: A Homeostasis-Based Amplification Mechanism

Studies of cellular AA homeostasis suggest there is a second amplification mechanism in which increases in the esterified membrane AA pool expand the amount of AA available for subsequent mobilization. This “AA pool expansion” is distinct from the acute free-AA increases described in Section 6.3 and reflects longer-term incorporation of exogenous AA into phospholipids. A larger intracellular AA pool would allow greater AA release upon stimulation and increase the capacity for downstream lipid-mediator formation, thereby elevating the gain of receptor-triggered signaling rather than providing a direct sensory input [28,40]. Within this framework, CaSR-evoked free-AA mobilization followed by lipid-mediator production offers a plausible route for sustaining intracellular activity and contributing to persistence in taste pathways without requiring AA to act as a taste ligand.

### 6.5. Implications: Reconciling Poultry Science Associations with Taste-Cell Biology

Reframing AA as a downstream lipid substrate rather than a direct taste ligand provides a coherent basis for interpreting poultry science associations. In this view, higher tissue AA levels primarily reflect a larger esterified membrane pool that can be mobilized after receptor activation and converted into lipid mediators that influence signaling persistence [2,4,28,40]. Embedding this receptor-to-lipid cascade within the hierarchical model separates food-level compositional correlations from intracellular mechanisms that shape intensity and persistence while still allowing interaction between the two levels.

## 7. A Hierarchical Model of Chicken Meat Palatability

**Figure 1:**
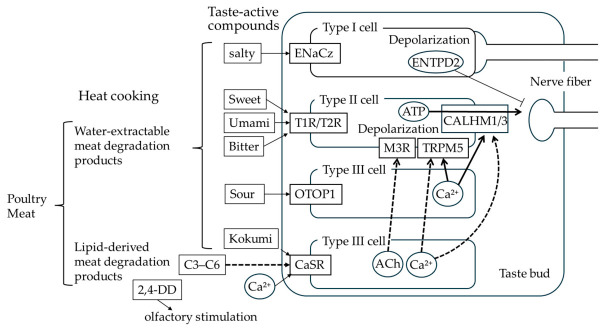
Hierarchical model of chicken meat palatability. Figure 1 states the central claim: AA-rich chicken is best understood as providing a substrate context that strengthens richness and persistence through (i) formation/transfer of water-phase lipid-derived modulators during cooking/handling and (ii) amplification modules in taste buds, rather than through direct “AA-as-tastant” detection. Food layer (left): breed/diet set the esterified AA/LA pools, and processing (cooking, storage oxidation, handling) determines which lipid-derived species form and whether they reach the oral aqueous fraction (soups/broths, meat juices, drip/cooking loss). Chemistry lane (left-to-middle): water-accessible low-molecular-weight oxidation products (notably short-chain aldehydes) are advanced as taste modulators (Section 3.1), while volatiles such as 2,4-DD are treated as a parallel aroma-side marker under defined cooking/capture protocols (Section 4; lane definitions in Section 3.1.3). Physiology layer (right): kokumi substances activate CaSR-expressing taste cells, and within-bud amplification can increase the gain and persistence of basic-taste signaling [6,7,36,39]. Solid lines indicate supported mechanisms; dashed lines indicate unverified links. Key within-bud wiring details remain unresolved (e.g., cell-type-resolved ACh release), so dashed arrows denote hypothesis-level links (Section 5.5.4 and Section 7). The model links matrix/aqueous-fraction chemistry (Section 3.1) and the volatile aroma lane (Section 4) to pathway-sensitive taste-bud readouts (Section 5.5.2) and the receptor-to-lipid cascade including downstream free AA mobilization (Section 6). Abbreviations: 2,4-DD, 2,4-decadienal; CaSR, calcium-sensing receptor; ACh, acetylcholine; M3R, muscarinic acetylcholine receptor subtype 3; ENaCz, epithelial sodium channel (salt-taste pathway); C3–C6, short-chain aldehydes (three to six carbons); T1R/T2R, sweet/umami and bitter taste receptors; CALHM1/3, calcium homeostasis modulator 1/3 (ATP-release channel complex); ENTPD2, ectonucleoside triphosphohydrolase 2 (ecto-ATPase).

Section 2, Section 3, Section 4, Section 5 and Section 6 integrate two complementary strands of evidence: food-level correlations linking AA-rich meat to enhanced sensory preference, and tastebud biology showing how kokumi-related pathways modulate intensity and persistence. Figure 1 synthesizes these strands into a hierarchical model that keeps correlation and mechanisms conceptually distinct while still enabling specific, testable connections between them—for example, phase transfer into aqueous fractions and pathway-sensitive tastebud readouts [2,4].

### 7.1. Upper Layer: Food Science and Poultry Science Perspectives

The upper level of the hierarchy contains food-composition and poultry science observations showing that meat with higher AA content is consistently associated with enhanced sensory preference in Japanese native chicken lines used for Jidori JAS production [2,4,5,6]. These associations, derived from compositional analyses and sensory evaluations, are robust but phenomenological: they indicate correlation rather than the sensory-physiology mechanisms that generate palatability. In the hierarchical model, AA content is therefore treated as a food-level compositional correlate that reflects the potential for enhanced palatability. Matrix and phase dependence remain testable hypotheses—for example, sensory differences may persist in fat-containing soups but diminish upon defatting—highlighting the need for matched cooking and sensory workflows when interpreting cross-study comparisons [2,4].

### 7.2. Lower Layer: Physiological and Cellular Taste Mechanisms

The lower layer of the hierarchy summarizes tastebud mechanisms that modulate intensity and persistence. Kokumi perception is framed as a receptor-mediated modulatory process centered on CaSR and within-bud amplification pathways that enhance tastant-evoked transmitter output [6,7,14,36]. Downstream of CaSR activation, intracellular signaling can mobilize AA from membrane stores and generate lipid mediators that tune the duration and gain of tastebud responses (Section 6.3 and Section 6.4) [28,40]. In this framework, AA functions as a downstream substrate contributing to amplification and lastingness rather than as an initiating taste stimulus, helping explain why kokumi-related qualities are often described in temporal terms and why food-level compositional correlations do not directly specify receptor activation [2,6,7,8].

### 7.3. Why the Two Layers Should Not Be Directly Connected by a Single Arrow

A central feature of the hierarchical model is that the food-level and taste-cell layers are not linked by a single causal arrow. Although meat AA content correlates with palatability, intact AA has not been shown to activate kokumi- or umami-related receptors such as CaSR or T1R1/T1R3. At the same time, AA can influence taste-cell output through downstream modulatory routes that are mechanistically distinct from primary-ligand receptor activation. Keeping these layers separate prevents correlation-level findings from being misinterpreted as receptor-level mechanisms. In this framework, higher AA content is treated as a food-level substrate context that may support stronger or more persistent modulation once CaSR-centered pathways are engaged rather than as evidence that AA itself functions as a primary CaSR ligand. This distinction preserves the conceptual integrity of the hierarchical model and avoids overextending food-level associations into unsupported mechanistic claims.

### 7.4. Hierarchical Integration Within the Proposed Model

Hierarchical integration allows the two layers to coexist without contradiction. The upper layer captures food-level correlations—such as the tendency for Jidori JAS chickens with higher AA content to be rated as more palatable—whereas the lower layer describes how modulation arises within tastebud physiology. Operational links between these layers can be evaluated by pairing food-level manipulations with pathway-sensitive readouts, including phase-dependent differences in soups or broths, targeted profiling of aqueous carbonyls, and perturbation assays of CaSR-linked amplification pathways. By keeping compositional associations distinct from taste-cell mechanisms, the model avoids reductionist single-compound explanations while clarifying which measurements belong to the food layer and which belong to the physiology layer, yielding a more coherent account of chicken meat palatability [2,3].

## 8. Complementary Pathways Contributing to Chicken Meat Palatability

Beyond the main CaSR-centered pathway, several additional mechanisms may contribute to perceived richness and persistence, including oral-fat-related cues, alternative kokumi-linked receptors, temporal coding within tastebuds, and central integrative processes [6,14]. More generally, dietary and food-borne lipids are well recognized as major contributors to food palatability through combined effects on flavor release, oral texture, and reward-related evaluation [29,41]. These pathways are treated as complementary as opposed to being in opposition to the primary model, and their value lies in how they can be experimentally separated. To maintain balance across mechanisms, the discriminating predictions below are framed in terms of a perturbation, an experimental readout, and the expected directional outcome.

(i) CaSR-centered kokumi amplification

Aroma cues are minimized (with nose-clip or taste-only designs), and ex vivo tastebud readouts such as ATP release or Ca^2+^ responses are measured during stimulation with basic tastants plus kokumi substances or PAM mixtures. Under these conditions, CaSR inhibition, reduced extracellular Ca^2+^, or muscarinic blockade should selectively diminish the enhancement—i.e., reduce gain or persistence—if CaSR-centered amplification is the operative mechanism [7,8,14,40].

(ii) Fat taste/lipid mouthfeel lane

The decisive manipulation is removal or perturbation of the lipid phase (via defatting or interference with fat-taste pathways such as CD36/FFAR) [42,43], paired with sensory descriptors emphasizing fat-related cues and physiological readouts where available. Effects in this lane should track the presence or disruption of the lipid phase, with little sensitivity to CaSR-specific blockade.

(iii) Aroma-dominant routes (volatile odorants)

Aroma-dominant explanations are evaluated by contrasting minimized-olfaction conditions (nose-clips and a controlled headspace) with conditions in which key volatiles are restored, alongside aroma-focused sensory descriptors and quantitative volatile profiling. If aroma drives the perceptual difference, effects should follow volatile odorants (e.g., 2,4-DD) and disappear when olfaction is restricted, consistent with a roast-aroma route rather than gustatory amplification.

### 8.1. Fat Taste and Lipid Mouthfeel

Fat taste (oleogustus) is increasingly recognized as a distinct oral modality mediated by receptors such as CD36 and FFARs, with supporting evidence from fatty-acid-responsive nerve fibers and cephalic-phase responses [42,43]. This pathway is conceptually independent of kokumi-related CaSR modulation. Although AA is a fatty acid, its proposed contribution to chicken meat palatability does not match the classical fat-taste mechanism for non-esterified long-chain fatty acids; the relevant concentrations, localization, and metabolic handling differ markedly from those typically implicated in oral fat detection. Fat-taste receptors are therefore treated as an independent contributor to richness and intensity rather than an explanation for AA-linked poultry science associations [4]. Human studies quantifying sensitivity to non-esterified fatty acids further support this complementary pathway, particularly in lipid-containing soups and broths [43]. A related, testable bridge is the fact that lipid-phase oxidation can generate aldehydes that act as candidate CaSR positive allosteric modulators, providing a route by which lipid fractions may enhance kokumi-like sensations without requiring AA to function as a primary ligand (Section 3.1.5 and Section 5.5.4) [14,25].

### 8.2. Kokumi Receptors Beyond CaSR

Several additional receptors have been proposed to be contributors to kokumi-like modulation. A representative example is GPRC6A, a notion supported by rodent studies in which low concentrations of L-ornithine enhanced taste-related behavioral and neural responses, with pharmacological antagonism and immunohistochemistry suggesting expression in subsets of tastebud cells [44]. However, evidence of these alternative receptors remains more limited than for CaSR, which currently has the most developed mechanistic support [7,8,36,38,39]. These candidates are therefore treated as complementary pathways rather than replacements for the CaSR-centered model.

### 8.3. Temporal and Aftertaste Mechanisms

The temporal dynamics pertaining to taste perception highlight that palatability depends not only on the initial response but also on how long signals persist after ingestion. Within tastebuds, intra-bud Ca^2+^ tone and prolonged CALHM1/3-mediated ATP release provide a plausible route for enhancing gain and persistence without altering primary taste quality. Kokumi perception is closely associated with continuity, and CaSR-linked sustained signaling offers one mechanistic basis for these temporal attributes. These temporal features align with consumer descriptions of lingering, rounded flavor in highly palatable chicken meat, positioning temporal coding as a complementary contributor to perceived richness [2,3,6,14,36,45].

### 8.4. Central Integration of Taste and Emotion

Even when peripheral taste mechanisms are well defined, perceived palatability ultimately depends on central integration, where taste, aroma, texture, and somatosensory inputs converge with learning and reward processes. Within this framework, peripheral kokumi- and lipid-related pathways are treated as contributors rather than complete explanations. CaSR-linked signaling and other peripheral mechanisms are therefore viewed as inputs that shape the quality, intensity, and persistence of sensory signals before they are integrated centrally. The hierarchical model positions these peripheral pathways as modulatory influences that inform—but do not determine—the centrally constructed experience of palatability.

### 8.5. Arachidonic-Acid-Derived Oxylipins as an Intracellular Modulation Layer

#### 8.5.1. Concept and Linkage to the Receptor-to-Lipid Cascade

One complementary concept is the idea that AA contributes to palatability through locally generated oxygenated lipid mediators (oxylipins) produced within tastebuds. In the hierarchical model, mobilized AA is enzymatically converted into short-lived mediators that modulate signaling gain and persistence, fitting within the receptor-to-lipid cascade outlined in Section 6.2 [28,40].

#### 8.5.2. Major AA-Derived Oxylipin Pathways (COX, LOX, and CYP)

AA mobilized within tastebuds can enter several enzymatic pathways that generate short-lived, locally acting oxylipins. The major routes include the cyclooxygenase (COX), lipoxygenase (LOX), and cytochrome P450 (CYP) pathways, which together define the core enzymatic map of arachidonic-acid metabolism discussed in this review [40].

- The cyclooxygenase (COX) pathway, featuring prostaglandins such as PGE_2_ and PGI_2_ [40];
- The lipoxygenase (LOX) pathway, featuring hydroxyeicosatetraenoic acids such as 12-HETE and 15-HETE [40];
- The cytochrome P450 (CYP) pathway, featuring epoxyeicosatetraenoic acids (EETs) and related epoxy/hydroxy metabolites [40].

#### 8.5.3. Proposed Modes of Action Within Tastebud Cells

Within tastebuds, oxylipins can act as short-range autocrine or paracrine modulators by engaging lipid-responsive receptors or influencing ion-channel activity. In this model, increased oxylipin signaling is expected to shift second-messenger dynamics, membrane excitability, and transmitter release toward more persistent responses, consistent with kokumi-like lastingness [28,40].

#### 8.5.4. Evidence Status and Testable Predictions

AA-derived oxylipins are positioned as an emerging intracellular modulation layer [40], and the model is most informative when expressed as testable predictions. If PGE_2_–EP signaling contributes to taste enhancement and persistence, then COX inhibition or EP-receptor antagonism should reduce kokumi-associated gain or lastingness in pathway-sensitive tastebud readouts under matched stimulation [40]. In parallel, if within-bud cholinergic modulation provides a required amplification step downstream of CaSR activation, atropine should attenuate CaSR-agonist-driven enhancement of sweet-evoked ATP output in isolated tastebuds, consistent with existing ex vivo evidence [7,14].

#### 8.5.5. Distinguishing Intracellular Oxylipins from Food-Chemistry and Fat-Taste Routes

The intracellular oxylipin pathway is distinct from two other lipid-related routes: (i) the food-chemistry route described in Section 3.1, which emphasizes water-extractable oxidation products generated during cooking, and (ii) the classical oral fat-sensing pathways outlined in Section 8.1. Together, these routes illustrate that lipid-related influences on palatability can arise at multiple levels—from food-generated, water-accessible modulators to intracellular lipid signaling that adjusts the gain and temporal profile of tastebud output [28,40].

#### 8.5.6. Minimal Experimental Roadmap

One minimal goal would be to quantify lipid-derived modulators—both oxylipins and low-molecular-weight carbonyls—in matched fat-containing versus defatted chicken soups and test causality by linking fractionation/reconstitution chemistry to tastebud outputs under targeted perturbations. These perturbations span CaSR inhibition, COX/EP-pathway blockade, muscarinic interference, and LOX-pathway manipulation, providing a unified framework for distinguishing food-generated modulators from intracellular lipid-signaling effects [7,8,13,14,25,27,28,40].

## 9. Implications for Poultry Research and Food Design

### 9.1. Implications and the Limits of Trait-Only Interpretations

The model has practical implications for poultry research and translating compositional differences into food design. Traditional meat-quality studies have emphasized growth performance, texture, and broad compositional traits [1,2], and AA has more recently been highlighted as a trait associated with palatability in Japanese native chicken lines used for Jidori JAS production [3,4]. The risk is that, without mechanistic context, AA will be treated as a single explanatory variable and discussion will stop at correlation. The present framework clarifies where AA can serve as a useful marker at the food layer and where mechanistic tests at the tastebud layer are required, enabling stronger, hypothesis-driven study designs.

Reframing AA as a downstream lipid precursor rather than a direct taste ligand provides a more actionable basis for poultry study design and interpretation [2,3]. AA remains a useful compositional marker (Section 2.2), but its practical value lies in supplying a larger mobilizable phospholipid reservoir. Once released through receptor-linked activation in tastebuds (Section 6.2), this pool can be converted into downstream lipid mediators that influence signaling persistence (Section 8.5) [2,3,27,28,40].

### 9.2. AA as a Downstream Substrate: Implications for Measurement and Study Design

Treating AA as a downstream substrate helps explain why higher tissue AA levels can correlate with greater palatability even if intact AA is not a primary taste ligand. The relevant variable is a larger, more responsive phospholipid AA reservoir that can be mobilized after kokumi-related receptor engagement and converted into lipid mediators influencing signaling persistence [2,3,27,28,40]. This shifts the emphasis toward measurable traits such as phospholipid AA proportion, phospholipid-to-neutral-lipid balance, and oxidation susceptibility under representative storage and cooking conditions. Excessive oxidation narrows the usable quality window by promoting the development of off-flavors [17,20,30], so a nuanced evaluation should jointly consider (i) a sufficiently large membrane AA reservoir for downstream mobilization and (ii) oxidative robustness rather than assuming that a single compositional marker will translate into improved palatability across products and workflows.

### 9.3. Food Design: Leveraging CaSR-Mediated Kokumi Without Increasing Salt, Sugar, or Fat Content

From a food-design perspective, CaSR-mediated kokumi mechanisms offer a route to enhancing perceived palatability without increasing salt, sugar, or fat content [6,7,36]. Ingredients rich in γ-glutamyl peptides or other CaSR activators can be incorporated into chicken matrices—including AA-rich products—to strengthen kokumi attributes such as thickness, mouthfulness, and continuity. This provides a basis for testable formulation hypotheses aimed at maintaining palatability under sodium- and energy-reduction constraints rather than serving as a product-positioning claim.

### 9.4. Practical Screening and Formulation Assays (Tastebud Output Readouts)

Recent mechanistic work has put forward practical screening strategies aligned with this framework. In tastebud preparations, CaSR agonists can amplify tastant-evoked ATP output through a within-bud cholinergic mechanism while not evoking output on their own [7,14]. These findings support a formulation logic focused on identifying kokumi-active ingredients that increase signal gain and persistence without increasing salt, sugar, or fat content. Operationally, candidate ingredients can be prioritized by conducting tastebud output assays and then testing whether responses are sensitive to CaSR inhibition and muscarinic blockade [7,14]. This provides an actionable bridge from receptor-level mechanisms to product development.

### 9.5. Processing and Product-Development Levers (Oxidation Kinetics and Phase Transfer)

If aqueous-phase lipid-derived modulators contribute to perceived richness and aftertaste, then several processing variables would become experimentally actionable. These include factors that shape oxidation kinetics (time–temperature history, oxygen exposure, antioxidants, and packaging) and factors that influence phase transfer into cooking loss or broth (fat separation, emulsification, and reheating). From a product-development perspective, these levers can be tuned within realistic workflows—such as chilled storage followed by reheating—while explicitly monitoring off-flavor formation to remain within an acceptable quality window [17,27,30].

## 10. Conclusions

The AA–palatability association in poultry is robust, but it is often interpreted in ways that imply a direct “AA-as-tastant” mechanism. A more biologically grounded interpretation is that AA functions as a substrate context whose effects on richness and persistence emerge from interactions between food chemistry and tastebud signaling.

The hierarchical model integrates two complementary routes: (i) a food-chemistry route in which controlled oxidation and cooking generate low-molecular-weight, water-extractable lipid-derived products that modulate taste, and (ii) a receptor-centered route in which CaSR-mediated kokumi signaling engages Ca^2+^ dynamics, mobilizes esterified AA, and recruits within-bud amplification modules. When linking chemistry to perception, it is essential to distinguish formation from aqueous-phase partitioning and adhere to evidence-aligned wording for cholinergic steps.

At the molecular level, candidate contributors are framed as a tiered hypothesis rather than a single-compound explanation: low-molecular-weight carbonyls (especially C5–C6 aldehydes) are the most evidence-supported gustatory modulators; (E,Z,Z)-2,4,7-tridecatrienal serves primarily as an AA-specific oxidation marker; and more polar/reactive secondary carbonyls are plausible drivers of quantitative AA>LA differences in the aqueous fraction. These points call for measuring chemistry directly in relevant aqueous fractions and linking concentration ranges to both enhancement and off-flavor onset.

Near-term validation logic aligns with the three food-focused predictions outlined in Food-Focused, Testable Predictions: (i) matrix/phase dependence; (ii) aqueous-phase carbonyl covariation; and (iii) pathway sensitivity.

Overall, this cross-disciplinary, hierarchical framing provides a practical basis for designing experiments and guiding processing and formulation strategies that connect measurable chemistry to tastebud-level mechanisms and sensory outcomes. Near-term validation logic is phase-sensitive (fat-containing vs. defatted systems), tracks measurable aqueous carbonyls, and weakens under pathway perturbations consistent with CaSR-centered amplification, as demonstrated in Section 7.4 and Section 8.5.

## Figures and Tables

**Table 1:** Solubility/partitioning-based working map for candidate lipid oxidation products in aqueous extracts (AA vs. LA).

Class (Typical Oxidation Stage)	Examples (LA-Derived, Illustrative)	Examples (AA-Derived, Illustrative)	Expected in Aqueous Extract?	AA Specificity
Parent PUFAs (substrates)	LA (non-esterified) and LA ethyl ester	AA (non-esterified) and AA ethyl ester	No	N/A
Primary oxidation products	LA hydroperoxides (HpODE; 9-/13-HpODE, generically)	AA hydroperoxides (HpETE, generically)	In low amounts	No
Low-molecular-weight carbonyls (secondary oxidation; β-scission products)	C3–C6 aldehydes such as pentanal and hexanal; small ketones	C3–C6 aldehydes (overlapping; e.g., hexanal) and small ketones (e.g., 1-octen-3-one)	Yes (partially)	No
Hydroxy-alkenals (secondary oxidation; electrophilic carbonyls)	LA-derived (representative): HNE (context-specific)	AA-derived (illustrative): AA can generate a broader set of hydroxy-alkenals, including HNE and HHE (context-specific)	It is possible	No
Dicarbonyls/dialdehydes (advanced oxidation)	MDA and other small dicarbonyls	MDA and other small dicarbonyls	Yes	No
Nonpolar unsaturated aldehydes/ketones (often aroma-potent)	2,4-DD (E,E/E,Z) and related long-chain unsaturated aldehydes	(E,Z,Z)-2,4,7-tridecatrienal (AA-specific); trans-4,5-epoxy-(E)-2-decenal; 2,4-DD; 1-octen-3-one; hexanal	No (except in cases of emulsion/protein-bound carryover)	Mixed. AA-specific: (E,Z,Z)-2,4,7-tridecatrienal. Not AA-specific: 2,4-DD and most other listed aldehydes/ketones.

This applies to the Kiyohara-type workflow (35 °C with 24 h autoxidation followed by aqueous extraction) [13,14]; examples are representative and intended to be a qualitative working map rather than evidence of quantitative dominance across meat systems. Solubility/partitioning rationale and caveats are discussed in Section 3.1.4. 2,4-DD is treated primarily as an aroma-side marker, and it is not considered a leading candidate for the taste-active aqueous fraction. Although 2,4-DD is a potent odorant (with a retronasal aroma), its low water solubility means it is not expected to be enriched as a free compound in aqueous extracts during typical defatting/partitioning steps. Therefore, within the hierarchical model, 2,4-DD is used mainly to track roast-aroma chemistry in the volatile lane. Any apparent presence in an “aqueous” fraction should be interpreted cautiously because it may reflect trace emulsified lipid and/or protein-bound carryover rather than a genuinely water-phase, tastebud-accessible ligand (see the general cautions in Section 3.1.4).

**Table 2:** Thigh-meat fatty-acid composition (LA, AA, and AA/LA) in broilers and Hinai-jidori [1].

Genotype	LA (%)	AA (%)	AA/LA
Broiler chicken (8 weeks of age)	17.06	1.42	0.083
Hinai-jidori chicken (22 weeks of age)	17.48	1.92	0.110

The proportion of AA was significantly higher in Hinai-jidori than in broiler chickens (*p* < 0.05).

**Table 3:** Qualitative mechanistic factors supporting the directional hypothesis *k_A_* > *k_L_*.

Mechanistic Factor	Directional Expectation (AA vs. LA)
β-scission propensity of hydroperoxide-derived alkoxyl radicals	Often argued to favor AA-derived HPETE fragmentation under heating (*workflow-specific*)
Effective ease of hydroperoxide decomposition (instability under heat/metal catalysis)	AA-derived hydroperoxides are commonly described as less stable, increasing fragmentation likelihood (*context-specific*)
Product-spectrum bias toward unsaturated aldehydes (dienal-rich channels)	AA oxidation can yield a broader unsaturated-carbonyl spectrum; LA often yields a more hexanal-dominant spectrum (*workflow-specific*)
Competing dominant channels/sinks	LA tends to channel strongly into C6 aldehydes, and AA can distribute into multiple carbonyls; both processes are subject to matrix quenching and loss

‘Workflow-specific’ indicates dependence on experimental or analytical procedures (e.g., heating or extraction methods). ‘Context-specific’ refers to dependence on interpretive or physiological settings (e.g., sensory vs. cellular responses).

## Data Availability

The data supporting the review are available from the corresponding author, H.T., upon reasonable request.

## Abbreviations

The following abbreviations are used in this manuscript:

JAS	Japanese Agricultural Standard
Jidori JAS	JAS-certified Jidori chicken
IMP	inosine 5′-monophosphate
AA	arachidonic acid
LA	linoleic acid
PUFA	polyunsaturated fatty acid
AAO	arachidonic-acid-enriched oil
CaSR	calcium-sensing receptor
GPCR	G protein-coupled receptor
VFT	Venus flytrap (domain)
HS-GC	headspace gas chromatography
HS-SPME-GC	headspace solid-phase microextraction gas chromatography
SPME	solid-phase microextraction
GC–MS	gas chromatography–mass spectrometry
LC–MS	liquid chromatography–mass spectrometry
LC–MS/MS	liquid chromatography–tandem mass spectrometry
PLA_2_	phospholipase A_2_
cPLA_2_	cytosolic phospholipase A_2_
TRPM5	transient receptor potential melastatin 5
2,4-DD	2,4-decadienal
HpODE	hydroperoxyoctadecadienoic acid
HpETE	hydroperoxyeicosatetraenoic acid
HNE	4-hydroxy-2-nonenal
HHE	4-hydroxy-2-hexenal (4-HHE)
C3–C6	short-chain aldehydes (three to six carbons)
MSG	monosodium glutamate
MDA	malondialdehyde
WOF	warmed-over flavor
ACh	acetylcholine
ChAT	choline acetyltransferase
M3R	muscarinic acetylcholine receptor subtype 3
ATP	adenosine triphosphate
CALHM1/3	calcium homeostasis modulator 1/3 (ATP-release channel complex)
ENTPD2	ectonucleoside triphosphohydrolase 2 (ecto-ATPase)
OTOP1	otopetrin 1 (proton channel implicated in sour taste)
ENaCz	epithelial sodium channel (salt-taste pathway)
PLC	phospholipase C
IP_3_	inositol 1,4,5-trisphosphate
COX	cyclooxygenase
EP	E prostanoid receptor
cAMP	cyclic adenosine monophosphate
PKA	protein kinase A
LOX	lipoxygenase
CYP	cytochrome P450
PGE_2_	prostaglandin E_2_
SIDA	stable isotope dilution analysis
PAM	positive allosteric modulator
CD36	cluster of differentiation 36 (fatty-acid translocase)
FFAR4 (GPR120)	free fatty-acid receptor 4
GPR120	G protein-coupled receptor 120 (also known as FFAR4)
FFAR1 (GPR40)	free fatty-acid receptor 1
GPR40	G protein-coupled receptor 40 (also known as FFAR1)
TAG	(E,Z,Z)-2,4,7-tridecatrienal
